# Follicular pancreatitis: Case report and literature review

**DOI:** 10.1097/MD.0000000000032837

**Published:** 2023-02-03

**Authors:** Wan-Sik Lee, Ki-Hyun Kim, Myung-Giun Noh, Young-Eun Joo

**Affiliations:** a Department of Internal Medicine, Chonnam National University Medical School, Gwangju, Korea; b Department of Pathology, Chonnam National University Medical School, Gwangju, Korea.

**Keywords:** chronic pancreatitis, follicular pancreatitis

## Abstract

Follicular pancreatitis is a very rare type of focal chronic pancreatitis and is often mistaken for pancreatic neoplasms. It is histologically characterized by extensive lymphoid follicular formation with reactive germinal centers.

**Patient concerns:**

A 50-year-old man was admitted to our hospital with 1-month history of epigastric pain. Contrast-enhanced computed tomography and magnetic resonance imaging revealed a 4.7 cm sized enhancing pancreatic head mass with anterior mesenteric soft tissue infiltration and superior mesenteric vein invasion. Endoscopic ultrasonography revealed an ill-defined hypoechoic mass in the head of the pancreas.

**Diagnoses:**

A laparoscopic surgical biopsy was performed. Hematoxylin-eosin staining showed the acini structure destruction within the pancreatic parenchyma and different-sized lymphoid follicles with reactive germinal centers around the duct. Immunohistochemical examination showed that cells were positive for the B-cell marker CD20, T-cell marker CD3, and slightly positive for IgG4. However, cells were negative for the B-cell marker Bcl-2. Follicular pancreatitis was confirmed based on the findings of histology and immunohistochemistry.

**Interventions:**

The patient was regularly followed without any specific treatment.

**Outcomes:**

Follow-up computed tomography revealed no change in the lesion 1 year after diagnosis.

**Lessons:**

To the best of our knowledge, this is the first case of follicular pancreatitis in Korea.

## 1. Introduction

Follicular pancreatitis is a very rare type of focal chronic pancreatitis and is often mistaken for pancreatic neoplasms. It is histologically characterized by extensive lymphoid follicular formation with reactive germinal centers. Patients with follicular pancreatitis are often asymptomatic and the lesions are incidentally discovered as a mass on radiologic imaging, suggestive of pancreatic cancer. Therefore, it is usually diagnosed retrospectively through a surgical approach.^[1–7]^

Herein, we report a case of a 50-year-old man with follicular pancreatitis, which was mistaken for pancreatic cancer, and the correct diagnosis was made through surgical biopsy, along with a review of the literature about this condition.

## 2. Case report

**Figure 1.**
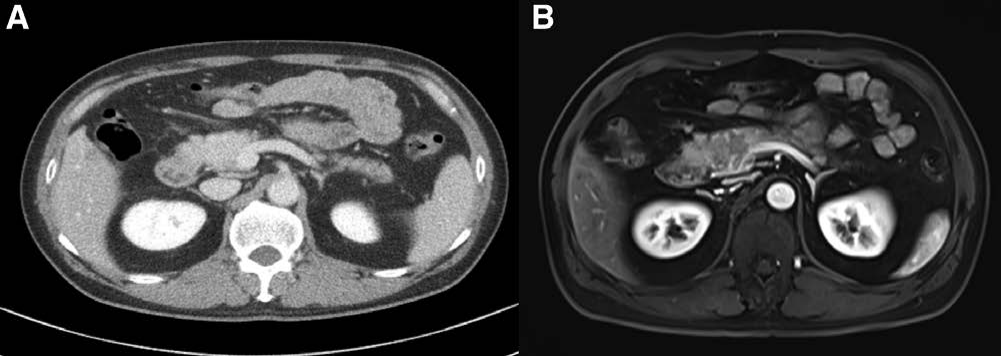
(A) Contrast enhanced abdomen and pelvis computed tomography shows a 4.7 cm sized enhancing mass with anterior mesenteric soft tissue infiltration in the head of the pancreas. (B) Pancreatic magnetic resonance imaging shows a pancreas head mass with superior mesenteric vein invasion.

**Figure 2.**
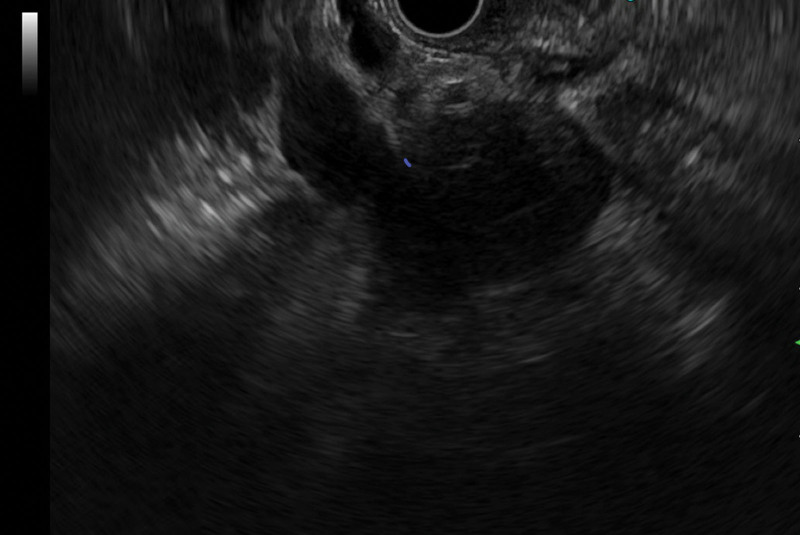
Endoscopic ultrasonogram shows an ill-defined hypoechoic mass in the head of the pancreas.

**Figure 3.**
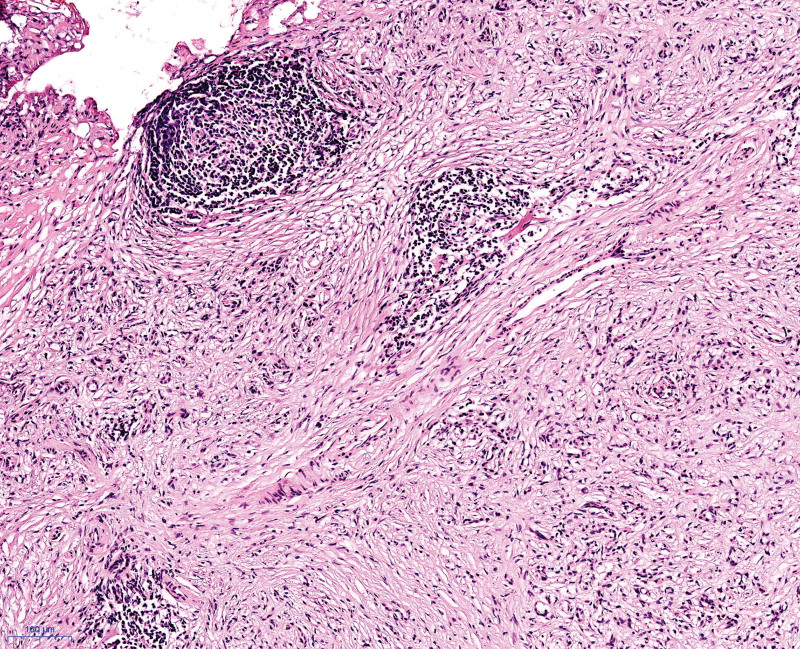
Routine histology, stained using hematoxylin-eosin shows the acini structure destruction at pancreas parenchyma and multiple sized lymphoid follicles with reactive germinal centers around duct.

**Figure 4.**
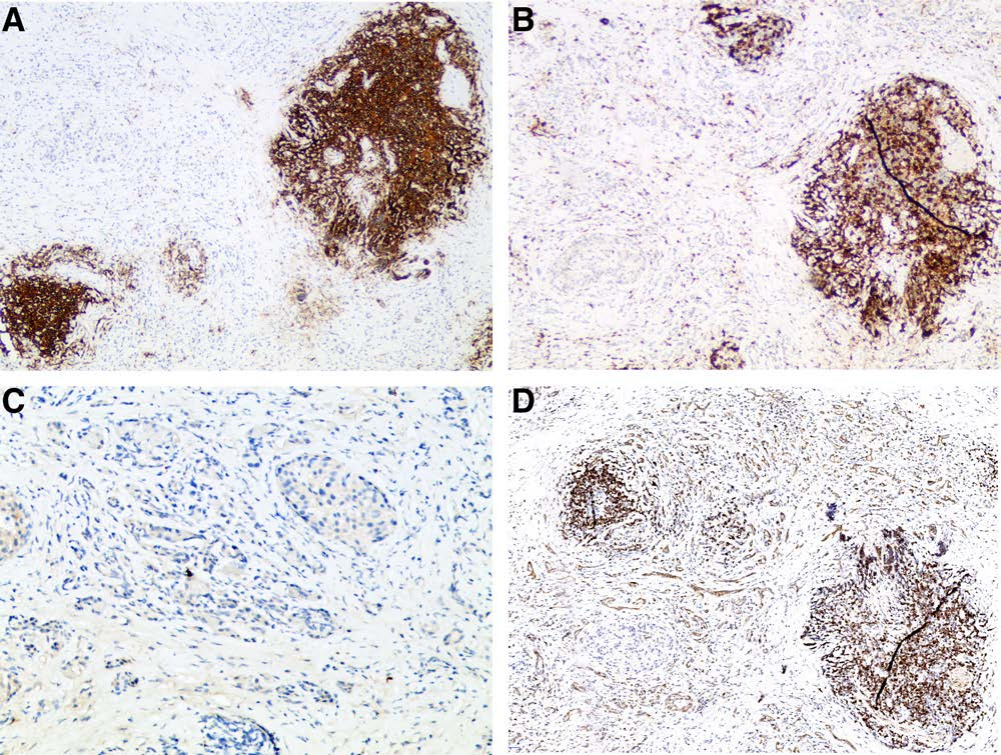
Immunohistochemical examinations show that cells are positive for the B-cell marker CD20 (A), T-cell marker CD3 (B), slightly positive for IgG4 (C), and negative for the B-cell marker Bcl-2 (D) (×400).

A 50-year-old man was admitted to our hospital with 1-month history of epigastric pain. He had no history of diabetes mellitus and hypertension. Furthermore, he had no history of alcohol drinking, although he was an ex-smoker with a history of 20 pack years. On admission, his vital signs were normal. There was tenderness in his epigastrium while other physical examinations were unremarkable. Laboratory examinations revealed the following findings: white blood cell count 6000/mm^3^ (normal range: 6000–10,000/mm^3^), hemoglobin 14.5 g/dL (normal range: 12–16 g/dL), platelet count 270,000/mm^3^ (normal range: 130,000–450,000/mm^3^), blood urea nitrogen 13.9 mg/dL (normal range: 8–23 mg/dL), creatinine 0.95 mg/dL (normal range: 0.5–1.3 mg/dL), serum albumin 4.5 g/dL (normal range: 3.0–5.0 g/dL), aspartate aminotransferase 21 U/L (normal range: 5–37 U/L), alanine aminotransferase 30 U/L (normal range: 5–40 U/L), alkaline phosphatase 124 U/L (normal range: 39–117 U/L), and lactate dehydrogenase 293 IU/L (normal range: 218–472 IU/L). Total bilirubin was 0.48 mg/dL with a direct fraction of 0.19 mg/dL (normal range: 0.2–1.2/0.05–0.3 mg/dL). Amylase was 159 U/L (normal range: 43–116 U/L) and lipase was 483 U/L (normal range: 7–60 U/L). Serum carcinoembryonic antigen (CEA) was within the normal limit. Carbohydrate antigen 19-9 was 68.75 U/mL (normal range: 0–39 U/mL). Contrast-enhanced abdominal and pelvic computed tomography (CT) revealed an enhancing mass (4.7 cm) with anterior mesenteric soft tissue infiltration in the head of the pancreas (Fig. 1A). Magnetic resonance imaging revealed a pancreatic head mass with superior mesenteric vein invasion (Fig. 1B). Contrast-enhanced neck and chest CT revealed negativity. Endoscopic ultrasound (EUS) revealed an ill-defined hypoechoic mass in the head of the pancreas (Fig. 2). Specimens from EUS-fine needle biopsy (EUS-FNB) showed several atypical lymphoid cell clusters with a high Ki-67 index. The correct diagnosis was difficult based on EUS-FNB. Thus, a laparoscopic surgical biopsy was performed for accurate histologic diagnosis. Hematoxylin-eosin staining showed the acini structure destruction within the pancreatic parenchyma and different-sized lymphoid follicles with reactive germinal centers around the duct (Fig. 3). Immunohistochemical examination showed that cells were positive for the B-cell marker CD20 (Fig. 4A), T-cell marker CD3 (Fig. 4B), and slightly positive for IgG4 (Fig. 4C). However, cells were negative for the B-cell marker Bcl-2 (Fig. 4D). Follicular pancreatitis was confirmed based on the findings of histology and immunohistochemistry. The patient was regularly followed without any specific treatment. He has been well and follow-up contrast-enhanced abdominal and pelvic CT revealed no change in the lesion 1 year after the surgical diagnosis. Informed consent was obtained from the patient for the purpose of publication.

## 3. Discussion

Follicular pancreatitis is an extremely rare form of mass-forming focal chronic pancreatitis. Because of its rarity, follicular pancreatitis is often mistaken for pancreatic cancer. The clinical manifestations, diagnosis, treatment, and prognosis of follicular pancreatitis have not been validated yet.^[1–7]^

Previous case reports^[2]^ suggest that follicular pancreatitis typically occurs in patients older than 50 years with a male predominance. The clinical presentation is often asymptomatic, although elevated levels of liver enzymes, abdominal pain, jaundice, malaise, and weight loss have been observed. The most commonly affected site is the pancreatic tail, followed by the head, body, and the uncinate process. A single pancreatic mass is most common on radiologic images, followed by multiple masses and no mass. On EUS, the appearance is usually a hypoechoic mass. In our case, follicular pancreatitis manifested as a single pancreatic head mass of hypoechoic nature in a 50-year-old man with abdominal pain. To the best of our knowledge, this is the first case of follicular pancreatitis in Korea.

Primary diagnosis of follicular pancreatitis is difficult since radiologic imaging findings strongly suggest the presence of pancreatic cancer. Most of the reported cases of follicular pancreatitis have been diagnosed retrospectively after surgical resection, sometimes with surgical complications.^[1–7]^ In our case, EUS-FNB was initially performed for the diagnosis; however, pancreatic cancer was not ruled out. Eventually, a laparoscopic surgical biopsy was performed for an accurate histologic diagnosis.

Histologically, follicular pancreatitis is characterized by extensive intra-pancreatic lymphoid follicles formation and dense lymphoplasmacytic infiltration. Furthermore, follicular pancreatitis is associated with diffuse or periductal Bcl-2 negative reactive germinal centers within lymphoid follicles and a low IgG4+ plasma cell count, which is similar to the findings of our case.^[6]^

The treatment of this disease is not well established yet. Most reported cases were diagnosed and treated by surgical resection, and there is no recurrence after surgical resection.^[1–7]^ One case partially responded to steroid therapy^[3]^; however, its efficacy remains unclear. Our case was regularly followed without any special treatment. Follow-up contrast-enhanced abdominal and pelvic CT revealed no change in the lesion 1 year after the surgical diagnosis. However, further follow-up is necessary to determine the long-term efficacy of this approach.

In summary, the dramatic differences in the treatment and prognosis of follicular pancreatitis and pancreatic cancer stress the importance of differential diagnosis. Therefore, despite its rarity, follicular pancreatitis should not be ruled out during the differential diagnosis of pancreatic cancer. Furthermore, a high index of clinical suspicion and an accurate histopathologic diagnosis is important for identifying patients with follicular pancreatitis. If an accurate histopathologic diagnosis is made before surgery, unnecessary surgery and surgical complications can be avoided.

## Author contributions

**Conceptualization:** Young Eun Joo.

**Investigation:** Wan-Sik Lee, Myung-Giun Noh.

**Resources:** Wan-Sik Lee, Ki-Hyun Kim, Myung-Giun Noh.

**Writing – original draft:** Wan-Sik Lee, Ki-Hyun Kim.

**Writing – review & editing:** Wan-Sik Lee, Young Eun Joo.
